# Changes in cortisol and cortisone in hair of pigs reared under heat stress conditions

**DOI:** 10.3389/fvets.2023.1156480

**Published:** 2023-07-18

**Authors:** Damián Escribano, Alexandra Contreras-Jodar, Marina López-Arjona, José Joaquín Cerón, Emma Fàbrega, Pau Aymerich, Antoni Dalmau

**Affiliations:** ^1^Interdisciplinary Laboratory of Clinical Analysis, Interlab-UMU, Regional Campus of International Excellence Campus Mare Nostrum, University of Murcia, Murcia, Spain; ^2^Department of Animal Production, Veterinary School, Regional Campus of International Excellence Campus Mare Nostrum, University of Murcia, Murcia, Spain; ^3^Animal Welfare Program, IRTA, Monells, Spain; ^4^Department of Animal and Food Science, Universitat Autònoma de Barcelona, Bellaterra, Spain; ^5^Vall Companys Group, Lleida, Spain

**Keywords:** animal welfare, corticosteroids, farm conditions, high temperatures, swine

## Abstract

Heat stress accounts for millions of dollars in losses for swine producers worldwide. The aim of the present study was to determine and evaluate cortisol and cortisone in hair as indicators of thermal stress in growing pigs reared under high environmental temperatures. The study was carried out in two independent batches of commercial crosses of Lean Duroc and Pietrain in trials 1 and 2, respectively, during the growing period (from 40 to 100 kg; 81 days in trial 1 and 77 days in trial 2) in the same commercial farm in Spain during the summers of 2020 and 2021. In both cases, four rooms were used. In Trial 1, Room 1 had cooling and 11 pigs per pen; Room 2 had no cooling and 13 pigs per pen; Room 3 had no cooling and 11 pigs per pen, and Room 4 had cooling and 13 pigs per pen. In Trial 2, Rooms 2 and 3 had cooling and rooms 1 and 4 had no cooling, and all of them had 13 pigs per pen. Mean THI value was higher (*p* < 0.0001) in rooms without cooling systems (75.0 trial 1; 74.9 trial 2) than with them (71.3 trial 1; 71.7 trial 2). A total of four pens per room (16 in total) was selected for analysis of hair corticoids and all pigs inside were sampled at the end of the study. Fifty percent of the pigs were males (castrated and intact in trial 1 and 2, respectively) and 50% females. In total, 44, 52, 44, and 52 pigs, respectively, were sampled in four rooms from the first trial and 52 for each of four rooms in Trial 2. Cortisol concentrations in hair did not show any significant change in relation to cooling-non-cooling in any trial. However, hair cortisone concentration was 172.3 pg./mg and 105.8 pg./mg less (*p* < 0.001) in pigs housed with cooling systems compared to those without them in Trial 1 and 2, respectively. In addition, the cortisone/cortisol ratio, which is an estimator of the activity of 11β-hydroxysteroid dehydrogenase (11β-HSD) type 2, was also greater in rooms without cooling than in rooms with cooling in both trials (*p* < 0.0001 and *p* = 0.0105 for Trials 1 and 2, respectively). In relation to the sex effect, the results showed greater levels in females than in castrated males both in cortisone and the cortisol/cortisone ratio while cortisol hair levels were greater in intact males than in females. Therefore, the use of cortisone and the estimation of 11β-HSD type 2 activity in hair is recommended to evaluate the chronic stress produced by high environmental conditions in pigs instead of using hair cortisol concentrations alone.

## 1. Introduction

Heat stress accounts for millions of dollars in losses for swine producers worldwide (1), and this problem is commonly due to high environmental temperatures, which usually persist for several months along the year in warm countries. In addition, climate change is causing global warming, with a higher frequency and duration of heat waves in warm countries and a rise in maximum temperatures and longer summers in colder ones. This global effect of the temperatures combined with the fact that pigs are used to being reared in intensive conditions with big populations of animals housed in high densities cause the risk of being subjected to heat stress to increase in pig production nowadays. On the other hand, in addition to ambient factors, it is important to take into account that pigs are much more sensitive to heat than are other species due to their physiological limitations. In fact, pigs cannot sweat and have little capacity for panting, in comparison to species like dogs, due to smaller lungs, a lesser degree of openness of the mouth and not being able to get the tongue out of the mouth, and they have a great accumulation of subcutaneous fat (2). Therefore, if they do not have a pond or bathing area (3), they have great limitations for cooling themselves. Furthermore, the use of an air conditioner is economically unviable for swine farms (2); consequently, in the mid-latitudes, pigs are kept predominantly in confined livestock buildings with a mechanical ventilation system (4) using evaporation pads. All of these facts make the validation essential of proper biomarkers of stress to be used in these conditions.

Blood cortisol is one of the main and most widely used biomarkers in pigs (5). Nevertheless, due to the need for handling the animals and the related stress response that acts as a confounding factor, in pig welfare studies (6) it is usually substituted by other non-invasive samples, such as saliva (7), feces (8), or hair (9). The use of hair samples for cortisol analysis has advantages, such as: simple handling, particular shipping and storage logistics is not needed, and it offers the possibility of a retrospective analysis of endogenous cortisol exposure (10, 11), which can be used to assess a chronic state of physiological stress. Cortisol is inactivated in hair by the action of 11β-hydroxysteroid dehydrogenase (11β-HSD) type 2 enzyme, converting it to cortisone whereas that 11β-HSD type 1 enzyme is involved in the reduction of the 11-keto group to alcohol, converting cortisone to cortisol and both isoforms are expressed in skin and hair (12). It has been concluded that the determination of cortisone in hair in species like sheep could be a promising alternative to assess animal welfare in chronic stress studies (13). However, to the authors’ knowledge, few studies have been conducted cortisone analysis in pig hair as a biomarker of chronic stress in this species.

In order to correctly assess cortisone concentrations, it is necessary to use a sensitive assay that is able to differentiate between cortisol and cortisone, avoiding cross-reactivity, to prevent erroneous results (14). In a recent study, two sensitive assays were developed and validated for the measurement of cortisol and cortisone, which also enabled the estimation of 11β-HSD type 2 activity, in the hair of sows during different phases of the reproductive cycle (15). In López-Arjona et al. (15), an increase in cortisone, possibly due to an increase in activity 11β-HSD type 2, was found in periods of high temperatures.

The aim of the present study is to determine and evaluate cortisone and cortisol as possible indicators of stress in the hair of growing pigs reared at high environmental temperature conditions in a commercial Spanish farm during two consecutive summers.

## 2. Methods

### 2.1. Management and husbandry of pigs

The study was carried out in the same commercial farm of pigs, located in Alcarràs, Lleida, Spain, during two consecutive trials, in summer 2020 and summer 2021. The experiment was approved by the Institutional Animal Care and Use Committee (IACUC) of IRTA, under Code 8348. The farm has two independent buildings, and each one has two independent rooms with a capacity of 728 pigs each (2,912 pigs in total). Each room consisted of a total of 56 pens allocated in four lines of 14 pens each. Between two lines of pens there was a corridor. Pens on the left side of the corridor allocated only castrated males (trial 1) or intact males (trial 2), and the right side allocated only females. Sexes were not mixed in the farm of the present study. Males from trial 1 were castrated in another commercial farm for sows and piglets following routine processes. This means that pigs were castrated at 3 days old without anesthesia or analgesia. The two first pens of each room were empty and used as hospital pens. The pigs of both trials were a commercial cross of Duroc Danbred x (Landrace x Largewhite) and reared in the farm. Pigs entered the farm 3 months old weighing 40 kg and they were reared until they reached 100 kg as a mean value in the first room at 6 months old. Once the first room achieved this weight, all rooms were sampled and the study finished. This occurred from July to September 2020 in Trial 1 and from June to August 2021 in Trial 2.

### 2.2. Experimental design

In Trial 1, two management strategies to reduce the consequences of heat stress in summer were tested: to provide a cooling system in Rooms 1 and 4, and to reduce the densities from 13 pigs per pen (0.68 m^2^/pig) to 11 pigs per pen (0.80 m^2^/pig) in Rooms 1 and 3. Consequently, pigs in each room were subjected to a specific treatment: Room 1 (cooling, low density), Room 2 (no cooling, high density), Room 3 (no cooling, low density) and Room 4 (cooling, high density). Trial 1 lasted 81 days. In Trial 2, only the use of cooling systems was maintained to reduce thermal stress, in this case used in Rooms 2 and 3, while Rooms 1 and 4 had no cooling. In Trial 2, all pens were maintained at the same density of 13 pigs per pen (0.68 m^2^/pig) Trial 2 lasted 77 days.

### 2.3. Cooling strategies

**Figure 1:**
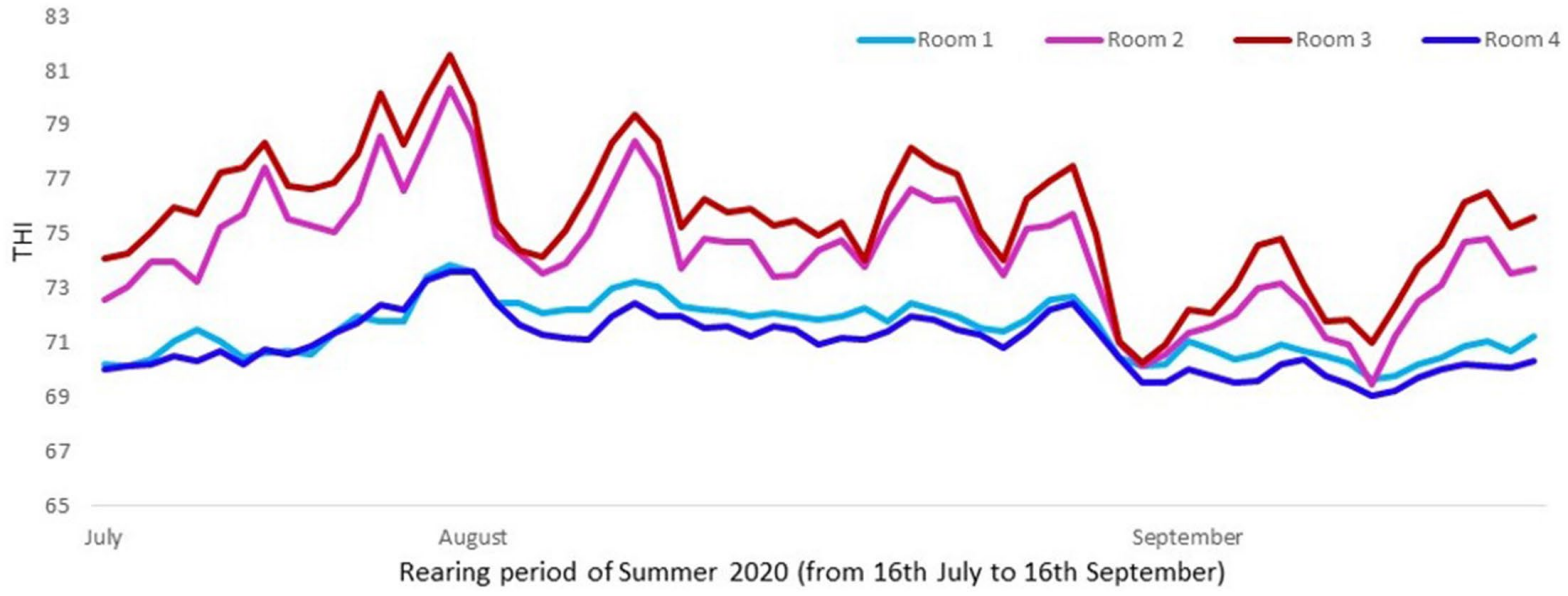
Mean daily THI values in rooms with and without cooling in Trial 1 (Room 1: cooling and 11 pigs per pen; Room 2: no cooling and 13 pigs per pen; Room 3: no cooling and 11 pigs per pen; and Room 4: cooling and 13 pigs per pen). Mean THI value was higher (*p* < 0.0001) in rooms without cooling systems (75.0) than with them (71.3).

**Figure 2:**
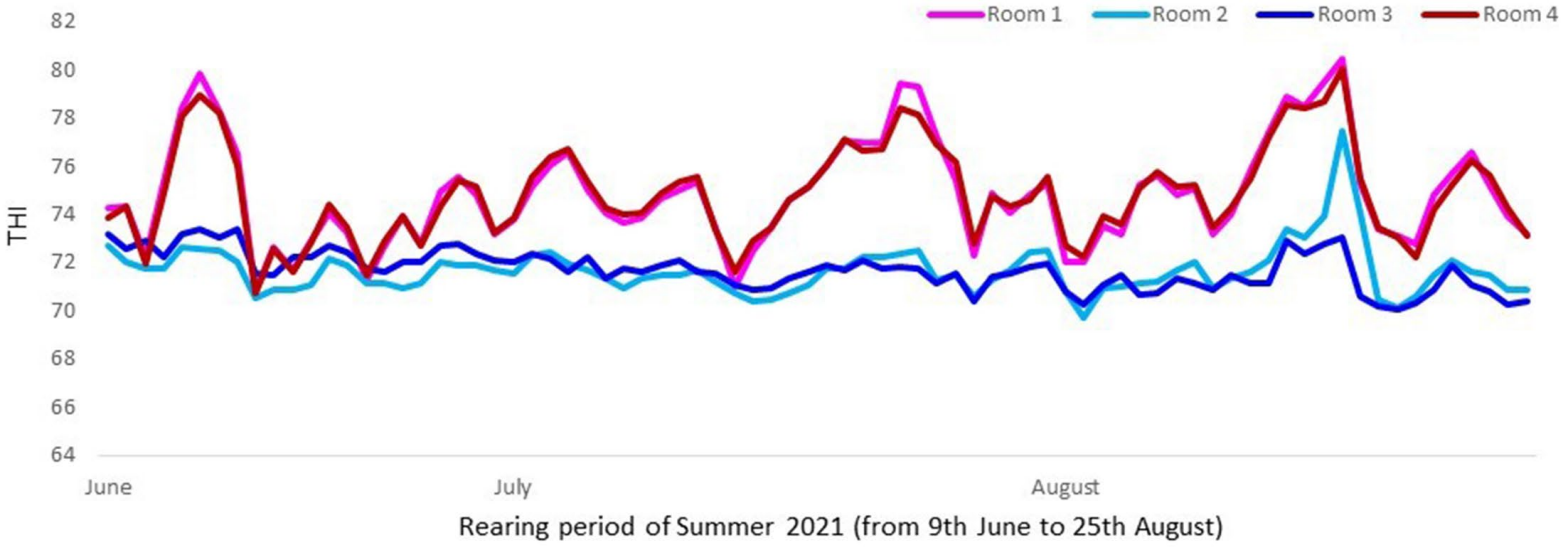
Mean daily THI values in rooms with (Room 2 and Room 3) and without cooling (Room 1 and Room 4) in Trial 2. Mean THI value was higher (*p* < 0.0001) in rooms without cooling systems (74.9) than with them (71.7).

The cooling system consisted in a mechanical evaporative cooling. This means that the unit used a fan to draw air through a wetted membrane, which provided a large surface area for the evaporation of water. Evaporative cooling exploits the fact that water will absorb a relatively large amount of heat in order to evaporate. The temperature of dry air can be dropped significantly through the phase transition of liquid water to water vapor (evaporation). For a better assessment of thermal conditions in pigs, instead of assessing just temperatures or humidity, the combination of both in an index (THI) was considered. The temperature was taken with a probe named “sonda temperature” (Exafan, Zaragoza, Spain) and humidity with a probe named “sonda humedad” (Exafan, Zaragoza, Spain). The THI was calculated according to The National Weather Service Central Region and Wegner et al. (16, 17) as follows: THI = [(1.8 T) + 32] –[0.55 (RH/100)] × [((1.8 T) + 32) – 58], where T is the air temperature in °C and RH the relative humidity in %, Figures 1, 2.

### 2.4. Sampling for hair and corticosteroid analysis

A total of four pens per room (16 in total per trial) were randomly selected and all pigs inside were sampled. Fifty percent of the pigs were males and 50% females. In total, 44, 52, 44, and 52 were sampled in Rooms 1; 2; 3; and 4, respectively in Trial 1, and 52 for each room in Trial 2. An area of around 10 cm × 10 cm located in the pig’s dorsal rump (10 cm from the tail) was selected at the end of the study for each pig, when pigs weighed 100 kg. Hair was collected by shaving close to the skin with clippers, trying not to remove the root of the hair, and avoiding inclusion of the hair follicle in the sample. Once sampled, hair was stored at −21°C inside hermetically sealed bags until analysis.

Cortisol and cortisone extraction from hair was performed as reported before by Daventport et al. (18). The hair samples were weighed (250 mg), placed in a polypropylene tube and covered with 5 ml of isopropanol. The tube was mixed at room temperature, centrifuged 1,500 *g* × 1 min and the isopropanol discarded. The samples were washed again with isopropanol and left at room temperature until completely dry. Next, the hair samples were cut into small pieces (around 5 mm) with scissors and 60 mg from each sample was weighed. The hair was placed in tubes with balls and pulverized to a fine powder in a homogenizer (Precellys Evolution homogeniser, Bertin Technologies; France). The pulverized hair was incubated with 1 ml of methanol for 18 h at room temperature with continuous gentle agitation for steroid extraction. Samples were then centrifuged 2000 *g* × 5 min and 0.6 mL of each methanol extract was aliquoted into a new Eppendorf tube. The samples were evaporated to dryness in a Speed Vac Concentrator (Concentrator 5301, Eppendorf). The dry extracts were reconstituted with 0.1 ml of phosphate buffer saline and stored at −80°C until analysis.

Cortisol and cortisone were measured in all samples with two sensitive assays based on AlphaLISA® technology (PerkinElmer) that were developed and validated previously for their use in pig hair (15) and the results expressed as pg./mg of hair. In addition, activity of 11β-hydroxysteroid dehydrogenase (11β-HSD) type 2 was estimated by cortisone/cortisol ratio. In the reported validation (15), the assays showed coefficients of variation <12% and the limits of blank were of 1.42 and 0.06 ng/ml and limits of quantification were 6.96 and 2.80 pg./ml for cortisol and cortisone, respectively.

### 2.5. Statistical analysis

Analyses were carried out with the Statistical Analysis System (SAS software, SAS Institute Inc.; Cary, NC, USA). Normality of residuals was checked through the Shapiro–Wilk test and QQ plots of residuals for each one of the dependent variables studied. The three variables studied, cortisol, cortisone and the cortisone/cortisol ratio met the normality assumption, so linear models by means of Proc Mixed were used. In Trial 1, the variables considered were cooling (yes or no), sex (males or female), density (11 pigs per pen or 13 pigs per pen) and all the interactions between factors. In the case of Trial 2, the variables considered were cooling (yes or no), sex (males or females) and the interaction between cooling and sex. In all cases, the pen of origin of the pigs was considered as a random effect. When significant differences were found, the least-square means of fixed effects (LSMEANS) adjusted to Tukey was used for multiple comparisons. In all cases, significance was fixed at *p* < 0.05.

## 3. Results

**Figure 3:**
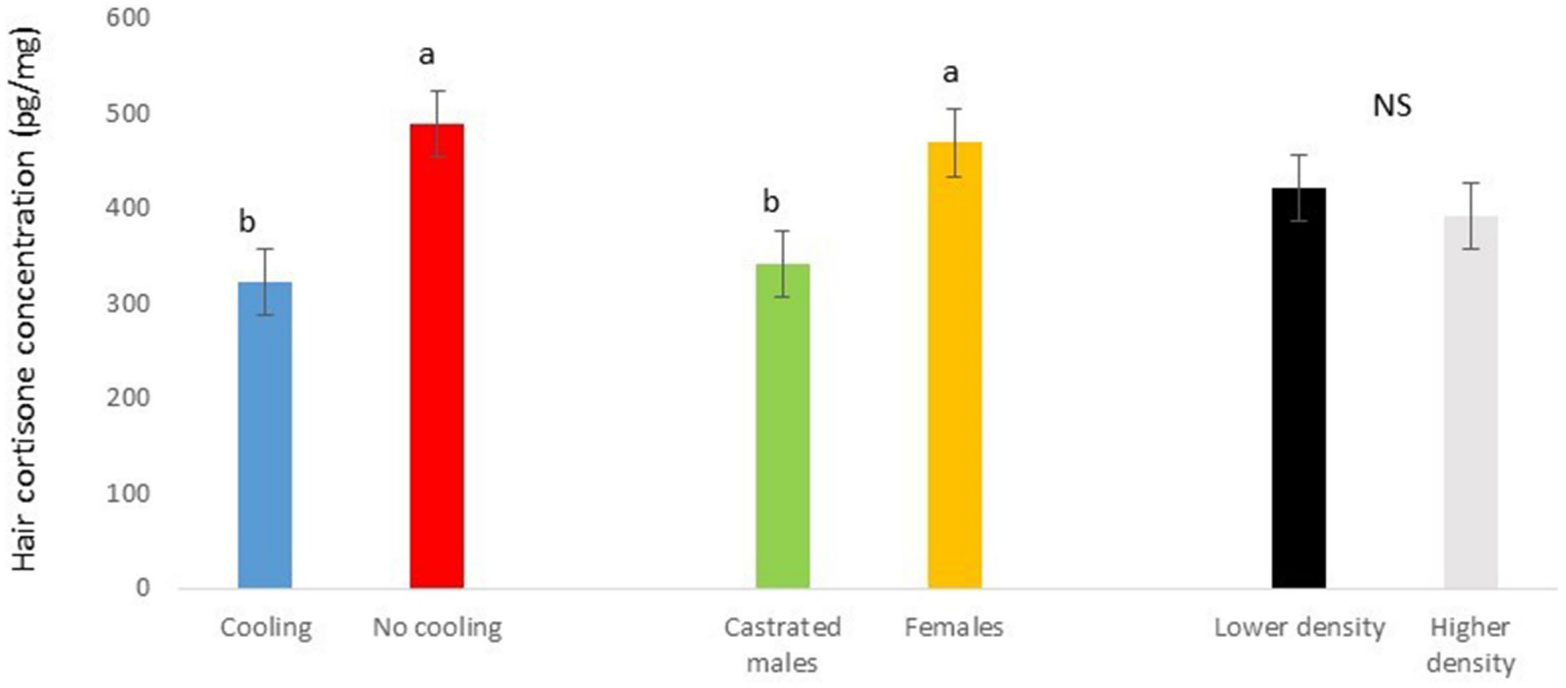
Hair cortisone concentration pg./mg of pigs during Trial 1 in relation to treatment (cooling vs. no cooling), sex (male vs. female) and density (11 or 13 pigs per pen). Different letters mean significant differences at *p* < 0.05; NS means not significant differences.

**Figure 4:**
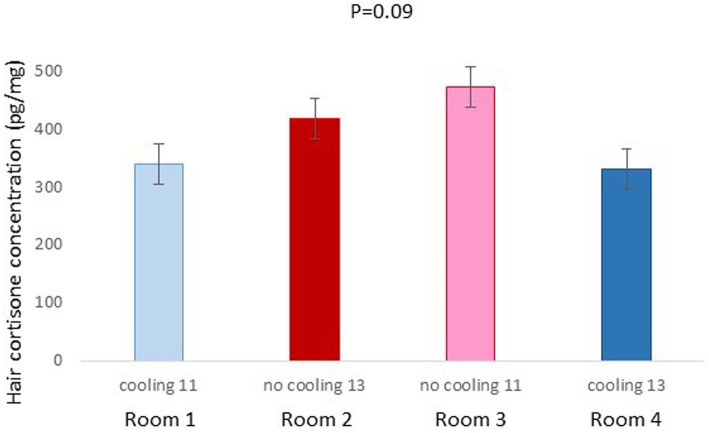
Hair cortisone concentration pg./mg of pigs of Trial 1 housed in the different rooms (*p* = 0.0904). Different letters mean significant differences at *p*=0.09.

**Figure 5:**
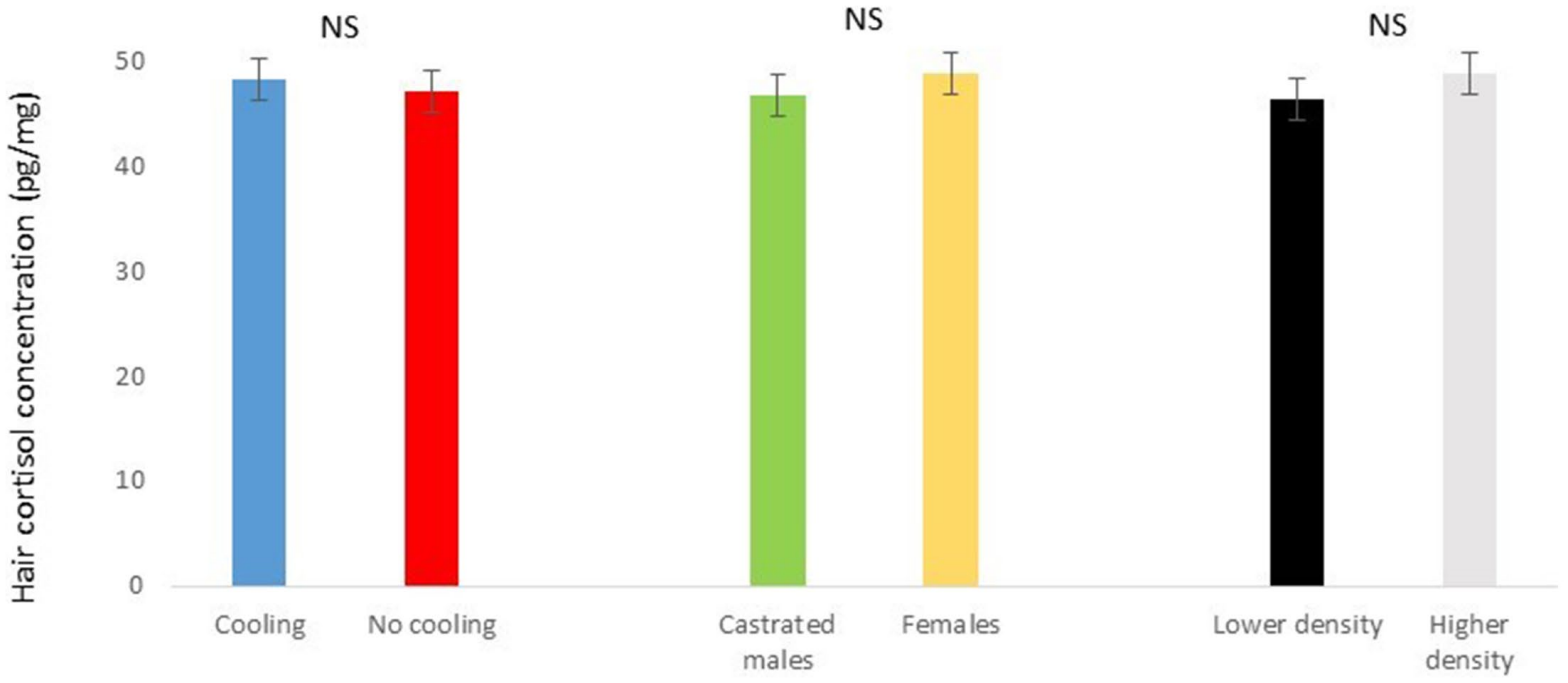
Hair cortisol concentration pg./mg of pigs during Trial 1 in relation to treatment (cooling vs. no cooling), sex (male vs. female) and density (11 or 13 pigs per pen). NS means not significant differences.

**Figure 6:**
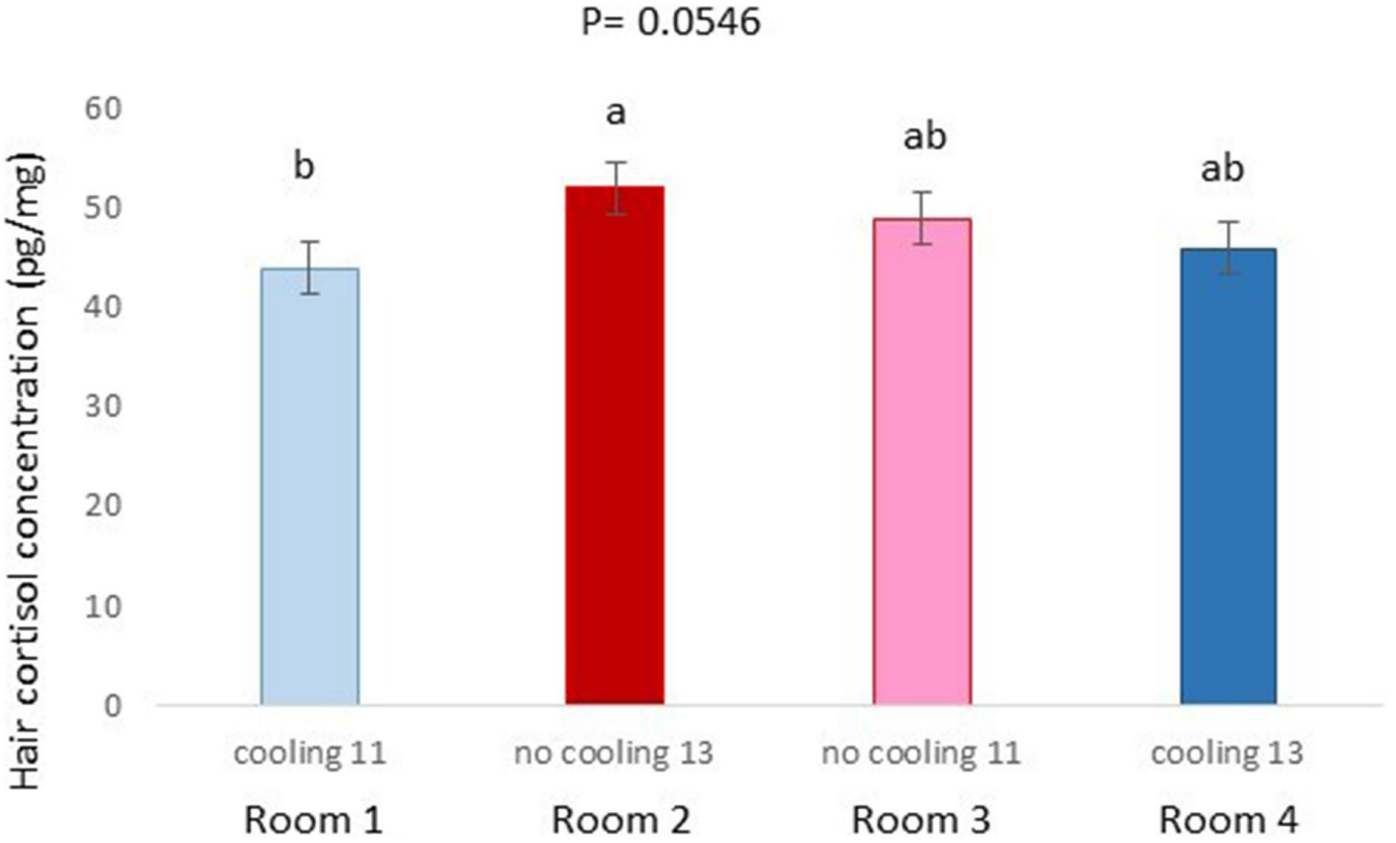
Hair cortisol concentration pg./mg of pigs of Trial 1 housed in the different rooms (*p* = 0.0546). Different letters mean significant differences at *p*=0.0546.

**Figure 7:**
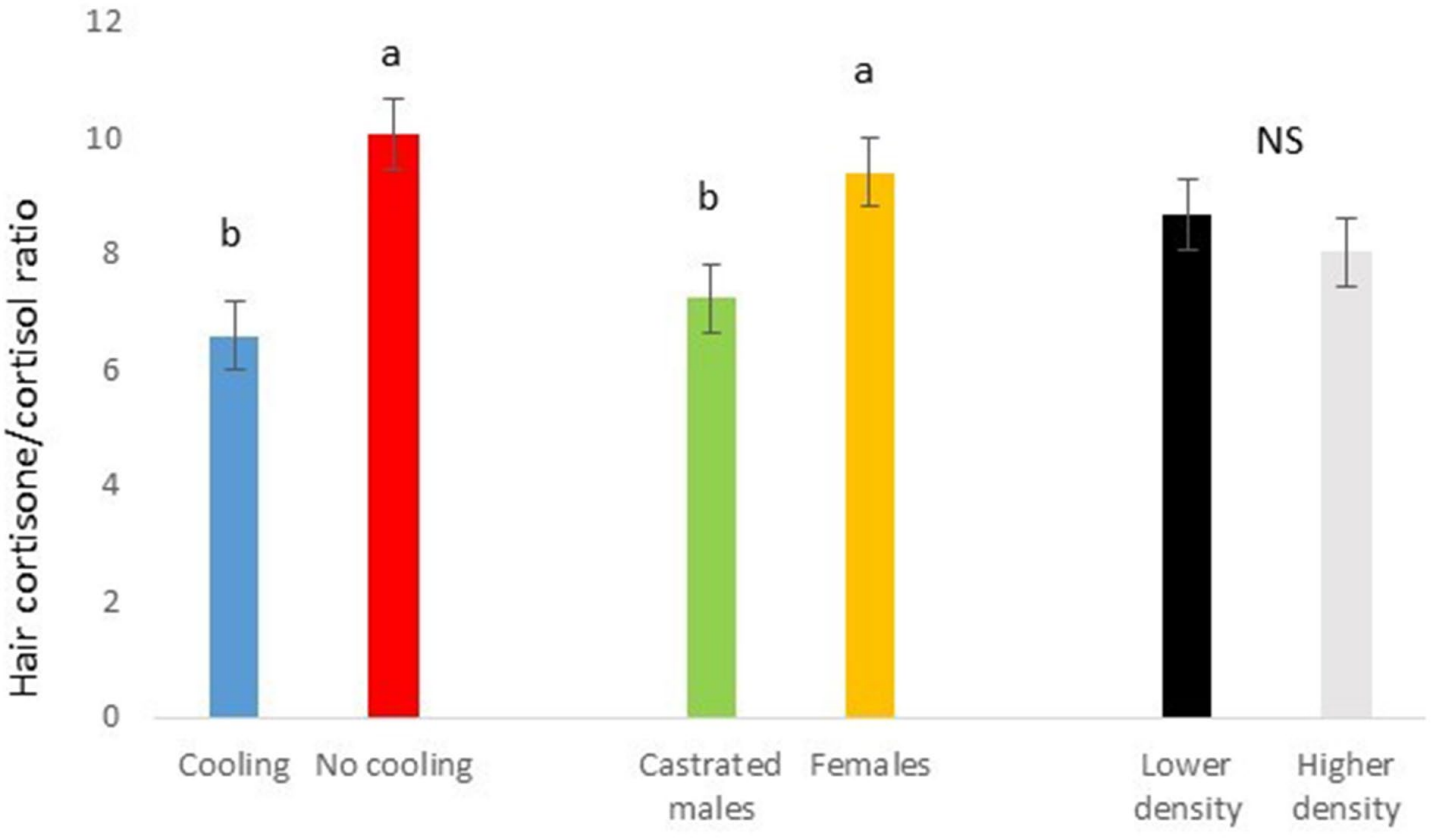
Cortisone/cortisol ratio in Trial 1 in relation to treatment (cooling vs. no cooling), sex (female vs. male) and density (13 vs. 11 pigs per pen). Different letters mean significant differences at *p* < 0.05; NS means not significant differences.

In Trial 1, for cortisone, a cooling effect (*p* = 0.0005; *F* = 12.4; DF = 1/176) and a sex effect (*p* = 0.0094; *F* = 6.89; DF = 1/176) were found, in addition to a trend for the interaction between cooling and density (*p* = 0.0905; *F* = 2.90; DF = 1/176). In fact, the concentration of cortisone in hair was greater in the pigs housed without cooling (hair cortisone concentration was 492.3 ± 34.46 pg./mg) than in pigs housed with cooling (hair cortisone concentration was 320 ± 34.46 pg./mg; Figure 3). In addition, this value was greater as well in females (hair cortisone concentration was 470 ± 34.42 pg./mg) than in castrated males (hair cortisone concentration was 342.7 ± 34.42 pg./mg; Figure 3). Finally, the trend shows how Room 3, the one reaching the highest mean THI (Figure 1) reached, as well, the maximum mean value for cortisone in hair (Figure 4, *p* = 0.0905). In Trial 1, for cortisol, no effect of treatment (cooling vs. no cooling), sex (males vs. female) or density (13 or 11 pigs per pen) was found, Figure 5, and just a trend for the interaction between cooling and density (*p* = 0.0546; *F* = 3.74; DF = 1/176) was found (Figure 6). For the cortisone/cortisol ratio, a cooling effect (*p* < 0.0001; *F* = 17.19; DF = 1/176) and a sex effect (*p* = 0.0112; *F* = 6.57; DF = 1/176) were found. In these cases, the cortisone/cortisol concentration ratio was greater in pigs reared without a cooling system (10.1 ± 0.60) compared to those reared with (6.6 ± 0.60) and, also, greater in females (9.4 ± 0.60) than in castrated males (7.3 ± 0.60; Figure 7).

**Figure 8:**
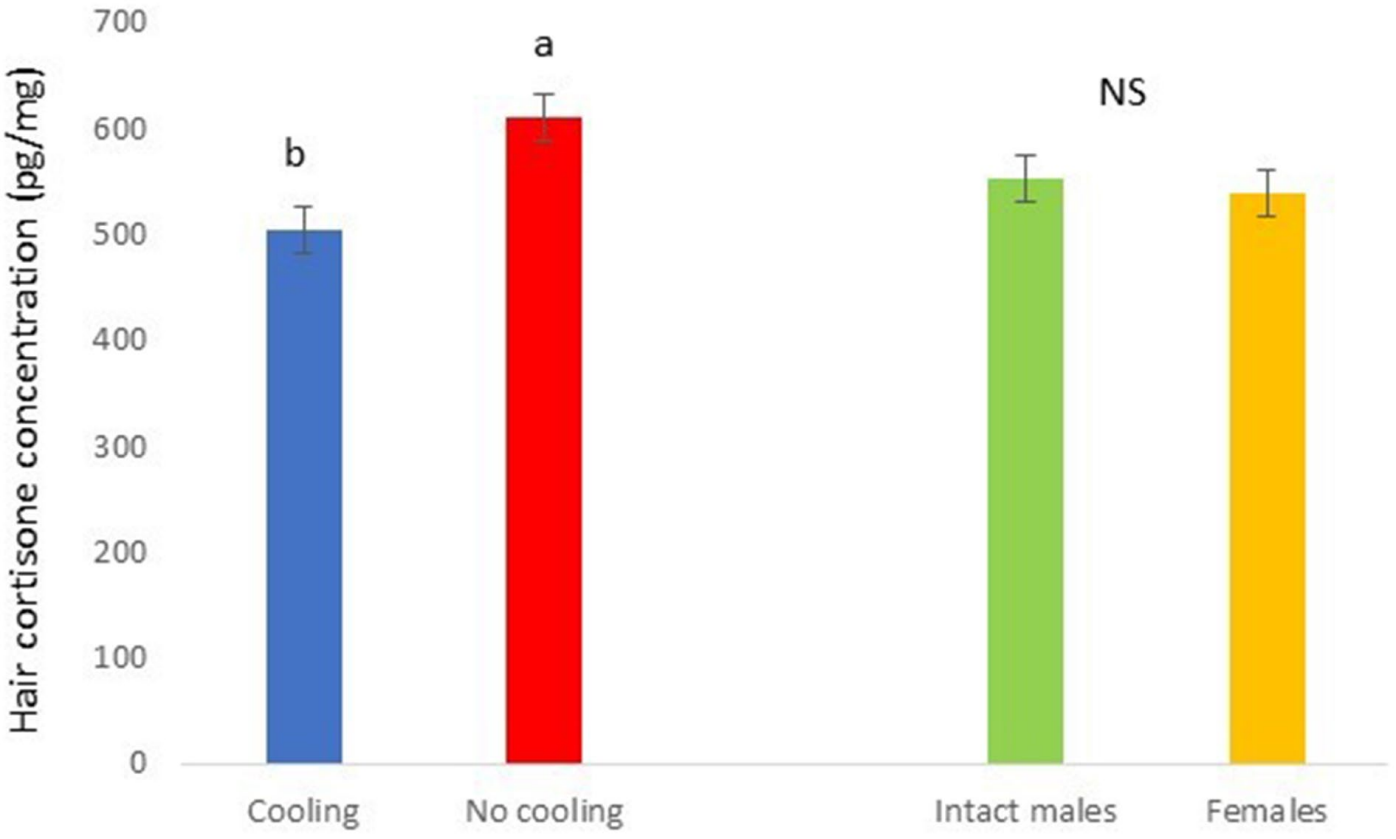
Hair cortisone concentration pg./mg of pigs during Trial 2 in relation to treatment (cooling vs. no cooling) and sex. Different letters mean significant differences at *p* < 0.05; NS means not significant differences.

**Figure 9:**
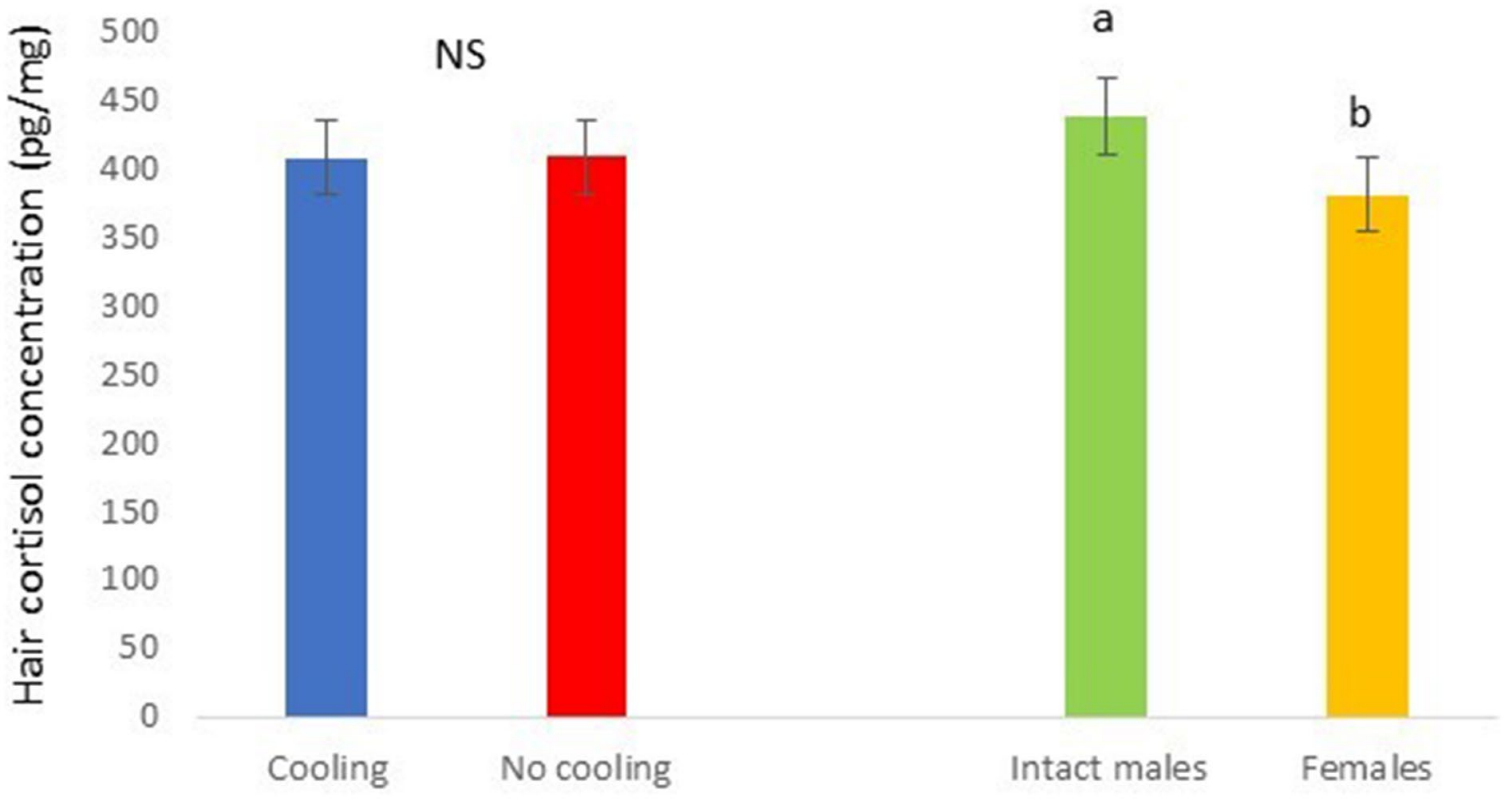
Hair cortisol concentration pg./mg of pigs during Trial 2 in relation to treatment (cooling vs. no cooling) and sex (male vs. female). Different letters mean significant differences at *p* < 0.05; NS means not significant differences.

**Figure 10:**
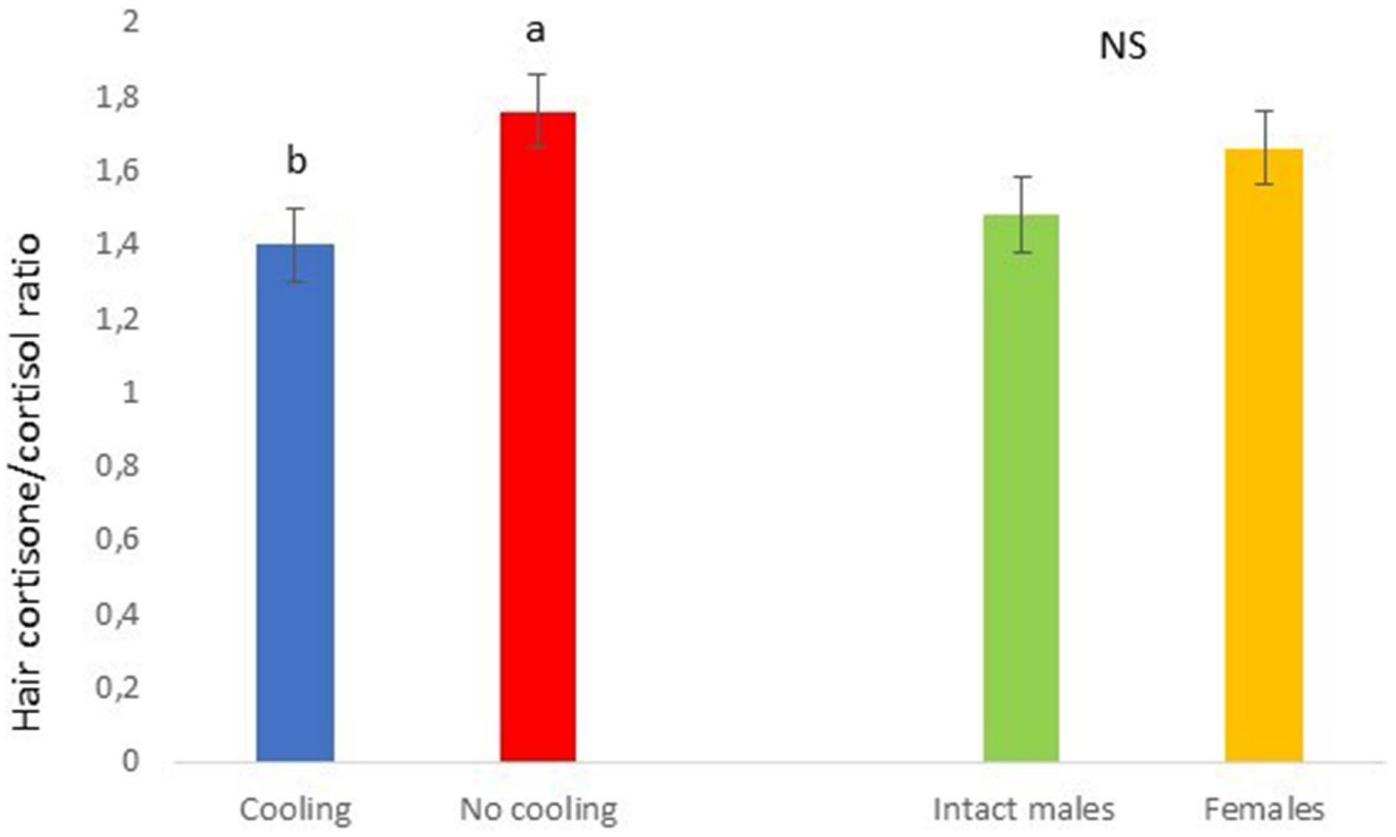
Cortisone/cortisol ratio in Trial 2 in relation to treatment (cooling vs. no cooling) and sex. Different letters mean significant differences at *p* < 0.05; NS means not significant differences.

In Trial 2, for cortisone a cooling effect was found (*p* = 0.0003; *F* = 13.85; DF = 1/183), with greater values in pigs within the rooms without cooling (hair cortisone concentration was 604.6 ± 22.68 pg./mg) than in rooms with cooling (hair cortisone concentration was 498.8 ± 22.28 pg./mg; Figure 8). For cortisol, only a sex effect was found (*p* = 0.0101; *F* = 6.76; DF = 1/183); with intact males having greater values of cortisol in hair (hair cortisol concentration was 439.2 ± 37.94 pg./mg) than with females (hair cortisol concentration was 382.1 ± 37.65 pg./mg; Figure 9). Finally, for the cortisone/cortisol ratio, a cooling effect was found (*p* = 0.0105; *F* = 6.68; DF = 1/183), where pigs reared in rooms without a cooling system (1.76 ± 0.109) had a greater ratio than those reared in rooms with it (1.39 ± 0.107) (Figure 10).

## 4. Discussion

In both trials the combination of temperature and humidity was significantly higher in rooms without cooling than in rooms with cooling, reaching a mean THI value of 75 in comparison to the 71.3 in rooms with cooling, which confirmed that pigs reared without a cooling system were in risk to be subjected to higher heat stress (17, 19). In fact, Wegner et al. (19) defined a threshold of 74 as a starting point based on heat stress for sows housed in closed insulated and ventilated barns in Germany. According to EFSA (20), in growing and finishing pigs, the thermoneutral zone varies between dry air temperatures of 15–28°C in a humidity range between 40 and 80%. In the present study the daily mean temperature reached 29, 30, 31, and 32°C (THI from 76 to 82) in rooms without cooling, while these values were not reached in rooms with cooling.

In both trials of the experiment, cortisone showed greater concentrations in the rooms without cooling than in rooms with cooling. In the first trial, the pigs without cooling had a hair cortisone concentration of 172.3 pg./mg greater than those with cooling, and in the second trial this difference was of 105.8 pg./mg. These differences are in line with the results shown by López-Arjona et al. (15), where, in sows that were at 5 days before farrowing during the warm season, the reported values of cortisone were 224.4 pg. per mg/hair greater than in sows at 5 days before farrowing during the colder months.

Cortisol concentration in hair did not show any significant difference in relation to cooling-non-cooling treatments for either of both trials. The fact that hair cortisol does not increase under heat stress conditions is in accordance with the results of López-Arjona et al. (15) in sows subjected to high temperatures. On the other hand, some authors consider that cortisol in hair is not really a good biomarker of stress. For example, Wiechers et al. (21) showed a lack of significant differences in cortisol in hair, when comparing farrowing systems with different situations of chronic stress, and the authors concluded that measuring hair cortisol might not be the most appropriate method for assessing stress in pigs. In other species, such as sheep, it was also found that hair cortisone levels were greater than was hair cortisol in a model of chronic stress (13).

According to the results of López-Arjona et al. (15), the results of the present study showed that with higher atmospheric temperature there is a greater change in hair cortisone concentrations and in the cortisone/cortisol ratio. A possible explanation of these results could be that at higher temperature the 11β-HSD type 2 presents a higher activity of action. In a kinetic study of 11β-HSD in rat submandibular salivary gland it was found that the NAD-dependent enzyme, the 11β-HSD type 2, increased its activity as it was subjected to more temperature, increasing exponentially up to 40°C temperature, while the activity of the enzyme NADPH-dependent, the 11β-HSD type 1, showed a slight increase (22). Although more studies are needed to clarify how both isoenzymes act in the case of hair tissue of pig, it could be postulated that this fact could also explain why no significant decreases were observed in cortisol, since an increase in the magnitude of action of 11β-HSD type 2 does not mean an inactivation of 11β-HSD type 1, cortisol production continues although in a reduced amount compared to the production of cortisone induced by 11β-HSD type 2 (23). In this line, the results of the cortisone/cortisol ratio indicates a lower activity of this enzyme in the rooms with cooling. In the first trial, the pigs with cooling had a 3.5 pg. cortisone/cortisol ratio per mg/hair less than those without cooling, and in the second trial this difference between cooling-non cooling was of 0.4 pg. cortisone/cortisol ratio per mg/hair. These results are also in line with those shown by López-Arjona et al. (15), where sows assessed during the warm season had a greater cortisone/cortisol ratio (7.97 pg), when compared to sows assessed during the colder months (0.19 pg). Overall, the results of the present study would indicate that it is preferable to use cortisone and the cortisone/cortisol ratio (an estimate of 11β-HSD type 2 activity) as indicators of chronic heat stress in pigs.

In relation to the sex effect, the results showed greater levels in females than in males both in cortisone and the cortisol/cortisone ratio, but only in the first trial, where castrated males were used. These results were not repeated in the second trial, where intact males were used and where a sex effect was found in cortisol hair levels, with greater levels in male than in female. To the authors’ knowledge, there are no studies on the influence of sex in cortisone or cortisol/cortisone ratio levels in hair between male and female pigs. However, the influence of sex in cortisol hair levels, although being inconsistent, may depend upon numerous factors such as different behavioral patterns, body condition and metabolism of gonadal steroids (24). In pigs, Trevisan et al. (25) showed that lean sows with poor body condition, had higher hair cortisol concentration compared with normal-weight sow. In other species such as cats, studies found higher hair cortisol concentration in aggressive animals, showing a significant positive correlation with the agonistic behavioral patterns (26) or lower concentration in castrated female than intact cats (26). In addition, the own sex-specific effects on regulation of HPA axis and the metabolism of gonadal steroids, appear to create different patterns of hair cortisol in male and female of nonhuman primates (27). Overall, more studies are needed to clarify how all those factors influence on hair cortisol concentration in porcine species, as in this study, intact males had higher levels of cortisol than females.

The results of the density effect in Trial 1 should be interpreted with caution, since in our report pigs at a higher density were thermally better (Figure 1) than were pigs at a lower density, and therefore thermal effects could influence the results. Therefore, although the animals in lower densities gained 3 kg more at the end of the growing period than animals in higher densities (data not shown), these differences could have been higher if the temperature in the rooms with high densities had not been cooler than in the rooms with lower densities. On the other hand, the combination of both cooling systems and lower densities resulted in a gain of 9 kg (data not shown). In addition, it is again a good example to consider in cases where specific stress factors (in this case a higher density) are assessed in conditions of high temperatures, as the use of hair cortisone and cortisol could result in better biomarkers than the use of cortisol alone, that showed just a trend for the combination of cooling systems and lower densities.

## 5. Conclusion

Overall, it could be concluded that pigs reared at high environmental temperatures show elevated hair cortisone without significant changes in hair cortisol concentration, plausible due to increased enzymatic activity of 11β-hydroxysteroid dehydrogenase type 2 under heat stress. These results suggest that measuring hair cortisone concentration may be a more sensitive biomarker of stress than hair cortisol concentration under stressfully high environmental temperatures.

## Data availability statement

The raw data supporting the conclusions of this article will be made available by the authors, without undue reservation.

## Ethics statement

The animal study was reviewed and approved by The Institutional Animal Care and Use Committee (IACUC) of IRTA, under Code 8348. Written informed consent was obtained from the owners for the participation of their animals in this study.

## Author contributions

DE, AC-J, JC, PA, and AD: conceptualization. DE and AD: writing—original draft preparation. AC-J, ML-A, JC, EF, and PA: writing—review and editing. JC and AD: supervision. EF, AD, and PA: project administration. AD and PA: funding acquisition. All authors have read and agreed to the published version of the manuscript.

## Funding

This study was included in Vall Companys, S.A.U.’s project WELFARE+ 8IDI-20210216 co-funded by Centre for the Development of Industrial Technology (CDTI), a Public Business Entity, answering to the Ministry of Science and Innovation, and the European Regional Development Fund (ERDF). DE was funded by the postdoctoral contract “Generational renewal to promote research” of the University of Murcia. ML-A (FJC2021-047105-I) by means of a post-doctoral fellowship, “Juan de la Cierva Formación,” supported by the “Ministerio de Ciencia e Innovación.”

## Conflict of interest

PA was employed by the company Vall Companys Group.

The remaining authors declare that the research was conducted in the absence of any commercial or financial relationships that could be construed as a potential conflict of interest.

## Publisher’s note

All claims expressed in this article are solely those of the authors and do not necessarily represent those of their affiliated organizations, or those of the publisher, the editors and the reviewers. Any product that may be evaluated in this article, or claim that may be made by its manufacturer, is not guaranteed or endorsed by the publisher.
